# *Trypanosoma cruzi* Growth Is Impaired by Oleoresin and Leaf Hydroalcoholic Extract from *Copaifera multijuga* in Human Trophoblast and Placental Explants

**DOI:** 10.3390/pathogens14080736

**Published:** 2025-07-25

**Authors:** Guilherme de Souza, Clara Peleteiro Teixeira, Joed Pires de Lima Júnior, Marcos Paulo Oliveira Almeida, Marina Paschoalino, Luana Carvalho Luz, Natália Carine Lima dos Santos, Rafael Martins de Oliveira, Izadora Santos Damasceno, Matheus Carvalho Barbosa, Guilherme Vieira Faria, Maria Anita Lemos Vasconcelos Ambrosio, Rodrigo Cassio Sola Veneziani, Jairo Kenupp Bastos, Angelica Oliveira Gomes, Rosiane Nascimento Alves, Carlos Henrique Gomes Martins, Samuel Cota Teixeira, Eloisa Amália Vieira Ferro, Bellisa Freitas Barbosa

**Affiliations:** 1 Laboratory of Immunophysiology of Reproduction, Institute of Biomedical Science, Universidade Federal de Uberlândia, Uberlândia 38405-318, MG, Brazil; guilhermesouza@ufu.br (G.d.S.); clapelet@gmail.com (C.P.T.); joed.lima@ufu.br (J.P.d.L.J.); marcospaulooliveiraalmeida@ufu.br (M.P.O.A.); marinapaschoalino@gmail.com (M.P.); luanacarvalholuz.28@gmail.com (L.C.L.); carine.natalia@ufu.br (N.C.L.d.S.); rafael_martinso@hotmail.com (R.M.d.O.); izadora.damasceno@ufu.br (I.S.D.); matcbio@gmail.com (M.C.B.); guifaria1825@gmail.com (G.V.F.); samuel.teixeira@ufu.br (S.C.T.); eloisa.ferro@ufu.br (E.A.V.F.); 2 Nucleus of Research in Technological and Exact Sciences, Universidade de Franca, Franca 14404-600, SP, Brazil; malvasconcelos@yahoo.com.br (M.A.L.V.A.); rodrigo.veneziani@unifran.edu.br (R.C.S.V.); 3 School of Pharmaceutical Sciences of Ribeirão Preto, Universidade de São Paulo, Ribeirão Preto 14040-903, SP, Brazil; jkbastos@fcfrp.usp.br; 4 Institute of Natural and Biological Sciences, Universidade Federal do Triângulo Mineiro, Uberaba 38025-015, MG, Brazil; angelica.gomes@uftm.edu.br; 5 Department of Agricultural and Natural Science, Universidade do Estado de Minas Gerais, Ituiutaba 38302-192, MG, Brazil; rosiane.alves@uemg.br; 6 Laboratory of Antimicrobial Testing, Institute of Biomedical Sciences, Universidade Federal de Uberlandia, Uberlandia 38405-318, MG, Brazil; carlosmartins2@ufu.br

**Keywords:** *Trypanosoma cruzi*, congenital Chagas disease, phytotherapeutic treatment, placenta

## Abstract

Congenital Chagas disease (CCD) is caused when *Trypanosoma cruzi* crosses the placental barrier during pregnancy and reaches the fetus, which can lead to serious consequences in the developing fetus. Current treatment is carried out with nifurtimox or benznidazole, but their effectiveness is limited, and they cause side effects, requiring the search for new therapeutic strategies. In this sense, many studies have demonstrated the potential of different compounds of the *Copaifera* genus in the control of parasitic diseases. Here, we aimed to evaluate the effect of oleoresin (OR) and leaf hydroalcoholic extract (LHE) of *Copaifera multijuga* on *Trypanosoma cruzi* infection in human villous trophoblast cells (BeWo line) and human placenta explants. Treatment with both compounds reduced invasion, proliferation, and release of trypomastigotes. Furthermore, OR and LHE affected the trypomastigotes and amastigote morphology, compromising their ability to invade and proliferate in BeWo cells, respectively. Also, treatment with OR decreased ROS production in infected BeWo cells, while LHE induced an increase. In addition, both compounds induced pro-inflammatory and anti-inflammatory cytokine production. In human placental explants, both compounds also decreased *T. cruzi* infection, in addition to inducing the production of pro-inflammatory cytokines. Thus, both OR and LHE of *C. multijuga* control *T. cruzi* infection at the human maternal–fetal interface, highlighting the possible therapeutic potential of these compounds for the treatment of CCD.

## 1. Introduction

Chagas disease is caused by the protozoan parasite *Trypanosoma cruzi*, and it is estimated that around 6 to 7 million people worldwide are infected, especially in Latin America, where the disease is endemic [1]. Transmission can occur through blood-feeding insects from the subfamily Triatominae, but other routes are possible, such as organ transplant, blood transfusion, and ingestion of contaminated food or beverages, as well as vertical transmission from the mother to the fetus, characterizing the congenital Chagas disease (CCD) [2].

CCD has become prominent, especially in non-endemic countries, where this form is responsible for around 22.5% of cases [3,4]. The congenital infection rate is around 5%, with the risk of transmission higher in endemic areas (5%) compared to non-endemic countries (3%) [3,5]. Although most of the infected newborns are asymptomatic, some will present with low birth weight, prematurity, and reduced Apgar scores (analysis of respiration, heart rate, reflexes, muscle tone, and skin color). In more severe cases, miscarriage, hepatosplenomegaly, respiratory distress, anemia, myocarditis, and meningoencephalitis may occur. In addition, certain newborns may present with life-threatening symptoms early on and are at risk of developing serious chronic conditions later in life [6,7,8].

During pregnancy, the maternal immune system creates a tolerogenic profile that is important for fetal growth, characterized by an anti-inflammatory profile and the production of important cytokines, such as interleukin (IL)-4 and IL-10 [9], as well as the macrophage migration inhibitory factor (MIF), IL-6, and IL-8, which are pro-inflammatory cytokines that play an important role during pregnancy [10,11]. Failure to maintain this immune response can compromise the progression of pregnancy and cause deleterious effects to the fetus. It is known that *T. cruzi* infection in the placenta affects the immune response, with high production of IL-1β, IL-6, IL-8, IL-10, and tumor necrosis factor (TNF-α) [12]. It is worth mentioning that IL-1β, IL-6, and TNF-α are associated with cell proliferation and trophoblast differentiation [13,14], which are related to the trophoblast defense mechanisms, known as epithelial turnover, and this can explain the low rate of *T. cruzi* transmission through the placenta [15,16]. On the other hand, TNF-α secretion can lead to a disruption of the placental barrier, leading to fetal death [13]. In this sense, the maternal immune system has the challenge of maintaining an immunological balance for fetal development and, at the same time, dealing with possible factors that can cause an imbalance in the immune response, such as an infection by *T. cruzi*.

Over the last 60 years, two classical drugs have been preferentially used to treat CD: nifurtimox (Nfz, Lampit^®,^, Leverkusen, Germany) and benznidazole (BNZ, Rochagan^®^, and Roche, Basel, Switzerland). Although highly effective in the acute stage, both drugs have some shortcomings, such as adverse side effects and lengthy treatment, and their effectiveness is shortened during the chronic stage [17]. Although treating infected women of childbearing age with these drugs can prevent congenital transmission in future pregnancies [18], treatment during pregnancy remains contraindicated due to the lack of safety data related to the fetus. This underscores the importance of Chagas screening during pregnancy and early detection of infection [19,20]. These limitations highlight the need for new therapeutic drugs that are effective and safe [21].

In this context, many studies have demonstrated the potential of natural products as biologically competent compounds to treat diseases. As an example, we turned our attention to the genus *Copaifera* spp. (Fabaceae family), also known as “copaiba”, which has been widely used and studied since it has shown anti-inflammatory, anticancer, antioxidant, and antimicrobial potential, among other applications [22,23,24,25]. Previous studies showed that *Copaifera* spp. can control infection by different protozoans, such as *Toxoplasma gondii* [26,27], *Leishmania* spp. [28,29], *Plasmodium* spp. [30], and *T. cruzi* [31,32].

Taking into consideration its popular use, here, we aimed to show the potential of oleoresin (OR) and leaf hydroalcoholic extract (LHE) from *Copaifera multijuga* against *T. cruzi* infection at the human maternal–fetal interface, reinforcing the potential of this genus as a source of molecules that can be used to design new drugs.

## 2. Materials and Methods

### 2.1. Plant Material

Both oleoresin and leaf hydroalcoholic extract from *C. multijuga* were kindly provided by Professor Carlos Henrique Gomes Martins from the Department of Microbiology (DEMIC), Institute of Biomedical Sciences (ICBIM), Universidade Federal de Uberlândia (UFU), Uberlândia, MG, Brazil.

Authorization for scientific studies involving plant species from Brazilian biodiversity was obtained from the Council for Authorization and Information on Biodiversity (SIBIO/ICMBio/MMA/BRAZIL) and Genetic Heritage Management (CGEN/MMA/BRAZIL) under authorization numbers 35143-1 and 010225/2014-5, respectively.

Authentic oleoresin was collected in the northern region of Brazil, in Manacapuru, Amazonas state, by Jonas J. M. da Silva. The exudate was obtained by drilling tree trunks with a 2-inch drill bit and collected in glass bottles. All the plant material collected was identified by Silvane Tavares Rodrigues, a voucher specimen (NID 03/2013 and 62/2013) was deposited in the Herbarium of the Brazilian Agricultural Research Corporation (Embrapa Eastern Amazon), and identified by direct comparison with authentic herbarium vouchers, of which a taxonomic identity certificate is available upon request.

Air-dried leaves of *C. multijuga* (200 g), dried at 40 °C for 48 h, were ground and exhaustively extracted by maceration with 1.2 L of ethanol/water (7:3) at room temperature for 48 h. After lyophilization, this yielded 50 g of hydroalcoholic extract of leaves [33].

### 2.2. Cell Culture and Parasite Maintenance

Human villous trophoblast cells (BeWo cell line) were obtained from the American Type Culture Collection (ATCC, Manassas, VA, USA). Cells were cultured in RPMI 1640 medium (Cultilab, Campinas, SP, Brazil) supplemented with 100 U/mL penicillin, 100 µg/mL streptomycin (Sigma Chemical Co., St Louis, MO, USA), and 10% fetal bovine serum (FBS) (Cultilab) in a humidified incubator at 37 °C with 5% CO_2_. Although BeWo cells are not of immune origin, several studies have demonstrated their ability to produce and secrete cytokines, such as IL-4 [34], IL-6 [35], IL-8 [35], IL-10 [36], and MIF [25], under various physiological and pathological conditions.

Tissue culture-derived trypomastigotes of *T. cruzi* (TCT, Y strain) were propagated in VERO cells in RPMI 1640 medium supplemented with 100 U/mL penicillin, 100 µg/mL streptomycin, and 2% FBS at 37 °C with 5% CO_2_.

### 2.3. Human Placenta Explants

Full-term human placentas (36 to 40 weeks of gestation) (*n* = 4) were collected from pregnant patients following elective cesarean sections at the Clinics Hospital of the Universidade Federal de Uberlândia (HC-UFU), MG, Brazil. Exclusion criteria included preeclampsia, hypertension, infectious disease, such as Chagas disease and toxoplasmosis, chronic renal disease, cardiac disease, connective tissue disease, diabetes, and other diseases that might affect the study outcomes. After collection, placenta tissues were washed in sterile phosphate-buffered saline (PBS) within 1 h to remove excess blood. Floating terminal chorionic villi, each containing five to seven free tips, were isolated, washed with 1x PBS, and placed individually into 96-well plates with 200 µL of RPMI 1640 medium supplemented with 5% FBS, penicillin (100 U/mL), and streptomycin (100 µg/mL) overnight at 37 °C with 5% CO_2_ [35]. Written informed consent was obtained from all participants included in this study, which had been previously approved by the Ethics Committee of Universidade Federal de Uberlândia, MG, Brazil, with approval number 7.407.162.

### 2.4. Cell Viability Assay

BeWo cell viability after treatment with oleoresin (OR) and leaf hydroalcoholic extract (LHE) was carried out by MTT assay [(3-(4,5-dimethylthiazol-2-yl)-2,5-diphenyltertrazolin bromide)], as described by [37].

BeWo cells (1 × 10^4^/100 µL) were seeded in 96-well plates in 5% FBS medium overnight at 37 °C with 5% CO_2_. Then, the cells were treated for 72 h with increasing concentration of OR and LHE from *C. multijuga* (ranging from 4 to 256 µg/mL) [25,26], 50 µM benznidazole (BZN) (LAFEPE, Recife, PE, Brazil) [32], 0.38% DMSO (vehicle present in the highest concentration of both compounds), and 5% FBS medium (negative control).

The cells were then incubated with MTT (5 mg/mL) diluted in a 10% FBS medium for 4 h at 37 °C with 5% CO_2_. After incubation, the resulting formazan crystals were dissolved in 10% sodium dodecyl sulfate (SDS, Sigma) and 50% N, N-dimethylformamide (DMF) (Sigma). Absorbance was measured at 570 nm using a microplate spectrophotometer (VersaMax ELISA Microplate Reader, Molecular Devices, Sunnyvale, CA, USA). Cell viability was expressed as a percentage of viable cells (cell viability %) by comparing the treatments with the cells treated only with 5% FBS medium (medium, 100% viability). Three independent experiments with eight replicates were carried out.

### 2.5. Trypomastigote Viability

*T. cruzi* trypomastigote viability treated with OR and LHE was also carried out by MTT assay, as previously described with minor modifications.

*T. cruzi* trypomastigotes (1 × 10^6^/50 μL) were seeded in 96-well plates in 5% FBS medium and immediately treated for 72 h with OR and LHE from *C. multijuga*, 50 µM benznidazole, and 5% FBS medium (negative control).

Afterwards, the TCTs were incubated with MTT (5 mg/mL) for 4 h at 37 °C with 5% CO_2_ and processed, as described previously. Trypomastigote viability was expressed as a percentage of viable parasites (trypomastigote viability%) by comparing the treatments with the parasites treated only with 5% FBS medium (medium, 100% viability). Three independent experiments with eight replicates were carried out.

### 2.6. T. cruzi Intracellular Proliferation

Intracellular proliferation of *T. cruzi* in BeWo cells treated with OR and LHE was assessed by counting intracellular amastigotes under a light microscope.

BeWo cells (4 × 10^4^/500 µL) were seeded onto 13 mm glass coverslips placed in 24-well plates in 5% FBS medium overnight at 37 °C with 5% CO_2_. The cells were infected with TCTY at a multiplicity of infection (MOI) of 1.3:1 (parasite/cell) for 3 h. After infection, cells were washed three times with 1x PBS to remove non-internalized parasites and then treated for 72 h with non-cytotoxic concentrations of OR (16 and 32 µg/mL), LHE (32 and 64 µg/mL), BZN (50 µM), and 5% FBS medium (control).

After treatment, the cells were washed with 1x PBS, fixed with pure methanol (Dinamica Química, São Paulo, SP, Brazil), and stained with Giemsa (Neon, Suzano, SP, Brazil) diluted in distilled water at a ratio of 1:20 for 20 min. Cells were then washed several times with 1x PBS and examined under a light microscope (Opton Microscopios, São Paulo, SP, Brazil). The infection rate was determined by counting the number of infected cells out of 200 randomly selected cells, and intracellular proliferation was assessed by counting the number of amastigotes within infected cells [32]. Three independent experiments, with three replicates, were carried out. The MOI chosen for this set of experiments (1.3:1) was used to avoid overinfection of host cells, which could interfere with accurate quantification.

### 2.7. Trypomastigote Release Assay

The trypomastigotes released in the supernatant were analyzed, as described by Kian and colleagues [38] with minor modifications.

BeWo cells (4 × 10^4^/500 µL) were seeded in 24-well plates in 5% FBS medium overnight at 37 °C with 5% CO_2_. The cells were infected with TCTY (MOI 5:1; parasite/cell) for 3 h. Next, the cells were washed three times with 1x PBS to remove non-internalized parasites and then treated for 72 h with non-cytotoxic concentrations of OR (16 and 32 µg/mL), LHE (32 and 64 µg/mL), BZN (50 µM), and 5% FBS medium (control).

Afterwards, the supernatant was collected and analyzed for the number of trypomastigotes released, while 5% FBS medium was added to the same plate to analyze the release of trypomastigotes on the following three days (96, 120, and 144 h), with the media being replaced every 24 h. The supernatants were centrifuged at 4000 rpm for 10 min and resuspended in 20 µL of medium, and then, the trypomastigotes were counted under a light microscope using a Neubauer chamber (Kasvi, Pinhais, PR, Brazil). Three independent experiments, with three replicates, were carried out. The MOI selected for this set of experiments (5:1) was standardized by us, as it ensures sufficient trypomastigote release at different time points, allowing reliable quantification without overwhelming the counting method.

### 2.8. Invasion and Proliferation of T. cruzi Pretreated with OR and LHE

The effect of parasite pretreatment with OR and LHE on its invasion and proliferation was also carried out by counting the intracellular amastigotes under a light microscope.

BeWo cells (4 × 10^4^/500 µL) were seeded onto 13 mm glass coverslips placed in 24-well plates in 5% FBS medium overnight at 37 °C with 5% CO_2_. The cells were infected for 3 h with TCTY (MOI 10:1 and 1.3:1 parasites/cell for invasion and proliferation experiments, respectively) that were previously pretreated for 1 h with OR (16 and 32 µg/mL), LHE (32 and 64 µg/mL), BZN (50 µM), or 5% FBS medium (control). Two different experimental protocols were carried out: (I) Invasion: after 3 h, the cells were washed three times with 1x PBS to remove non-internalized parasites and then processed to Giemsa stain; (II) Proliferation: after 3 h, the cells were washed three times with 1x PBS to remove non-internalized parasites, and the cells were treated with 5% FBS medium for additional 72 h and then processed to Giemsa stain, as previously described.

For both situations (invasion and proliferation), the cells were examined under a light microscope (Opton Microscopios, SP, Brazil) to determine the infection rate (number of infected cells/200 cells counted randomly) and the *T. cruzi* intracellular proliferation (number of amastigotes observed in infected cells) [32]. Two independent experiments, with four replicates, were carried out. For the invasion assay, a MOI of 10:1 was used to ensure a sufficient number of parasites to infect the host cells within a short incubation period.

### 2.9. Reactive Oxygen Species (ROS) Production

ROS production was carried out using a 2′,7′-dichlorodihydrofluorescein diacetate probe (H2DCF-DA, Invitrogen, catalog number: D399) to form the highly fluorescent compound 2′,7′-dichlorofluorescein (DCF).

BeWo cells (1 × 10^4^/100 µL) were seeded in a 96-well black clear-bottom plate in 5% FBS medium overnight at 37 °C with 5% CO_2_. The cells were infected with TCTY (MOI 1.3:1; parasite/cell) for 3 h. Next, the cells were washed three times with 1x PBS to remove non-internalized parasites and then treated or not for 72 h with non-cytotoxic concentrations of OR (16 and 32 µg/mL), LHE (32 and 64 µg/mL), and BZN (50 µM). As controls, the cells were uninfected or infected and treated with 5% FBS medium.

Afterwards, the supernatants were collected and stored at −80 °C for cytokine analysis. The cells were then washed with 1x PBS and incubated with the H2DCF-DA probe (10 µM diluted in 1x PBS with 10% FBS) for 45 min at 37 °C with 5% CO_2_, protected from light. Following incubation, the probe was removed, and the cells were washed with 1x PBS and incubated with PBS with 10% FBS for fluorescence reading. The mean fluorescence intensity of DCF was measured using a GloMax Explorer microplate reader (Promega, Madison, WI, USA) at excitation and emission wavelengths of 488 nm and 522 nm, respectively. Three independent experiments with eight replicates were carried out.

### 2.10. Morphological Analysis of Trypomastigotes and Amastigotes

The trypomastigote morphology was analyzed by scanning electron microscopy (SEM) [32]. *T. cruzi* trypomastigotes (1 × 10^7^/1000 µL) were treated with OR (32 μg/mL) or LHE (64 μg/mL), BZN (50 μM), or 5% FBS for 1 h. Next, the parasites were centrifuged at 4000 rpm for 10 min, washed 1x with sodium cacodylate buffer, and fixed in Karnovsky’s solution containing 2% paraformaldehyde and 2.5% glutaraldehyde in 0.1 M sodium cacodylate buffer for 2 h. Then, the parasites were washed with sodium cacodylate buffer and incubated with 1% osmium tetroxide (OsO_4_) for 1 h. Afterwards, the parasites were washed twice with sodium cacodylate buffer, and 10 μL of parasite was added to circular coverslips and left overnight at room temperature to dry. Subsequently, the coverslips with parasites were dehydrated in increasing concentrations of ethanol (50, 70, 80, 90, 95, and 100%, 5 min each), and a thin layer of gold was applied to the samples and then analyzed under a scanning electron microscope (Tescan, VEJA 3 LMU, TESCAN Orsay Holding, Brno, Czech Republic). One experiment with three replicates was carried out, and around 10–15 pictures were taken from each condition.

Also, we analyze the amastigote morphology by transmission electron microscopy (TEM). BeWo cells (1 × 10^6^/500 µL) were seeded in 24-well plates in 5% FBS medium overnight at 37 °C with 5% CO_2_. The cells were infected with TCTY (MOI 1.3:1; parasite/cell) for 3 h. Next, the cells were washed three times with 1x PBS to remove non-internalized parasites and then treated for 72 h with OR (16 and 32 µg/mL), LHE (32 and 64 µg/mL), BZN (50 µM), and 5% FBS medium (control). After treatment, the cells were collected, centrifuged at 1500 rpm for 5 min, and fixed in Karnovsky’s solution containing 2% paraformaldehyde and 2.5% glutaraldehyde in 0.1 M sodium cacodylate buffer for 24 h. Next, the cells were incubated with OsO_4_ for 1 h and treated with potassium ferrocyanide for 30 min, dehydrated in increasing concentrations of ethanol (50, 70, 80, 90, 95, and 100%) and 100% propylene oxide, and then embedded in Epon resin. Ultrathin sections obtained were stained with 3% uranyl acetate and 1% lead citrate and then analyzed using a transmission electron microscope (Hitachi HT7700, Hitachi High-Tech Corporation, Tokyo, Japan). One experiment, with three replicates, was carried out.

### 2.11. Viability of Human Placenta Explants Treated with OR and LHE

The viability of human placenta explants treated with OR and LHE from *C*. *multijuga* was evaluated by MTT assay and histological analysis [25].

Human placenta explants were prepared as previously described and cultured in 96-well plates in 5% FBS medium overnight at 37 °C with 5% CO_2_. Next, the villous explants were treated for 72 h with increasing concentrations of OR and LHE from *C. multijuga* (ranging from 4 to 256 µg/mL), 50 µM benznidazole (BZN), and 5% FBS medium (negative control). After treatment, the supernatants were collected and stored at −80 °C for later cytokine analysis, and the villous explants were submitted to the MTT assay, as previously described. Villous viability was expressed as the percentage viability of villous explants in relation to explants treated only with medium (medium, 100% viability). Additionally, treated and untreated villi were collected for morphological analysis using 5% hematoxylin and 0.1% eosin staining [25,32]. Two independent experiments, with eight replicates, were carried out.

### 2.12. T. cruzi Proliferation in Human Placenta Explants Treated with OR and LHE

*T. cruzi* proliferation in human placenta explants treated with OR and LHE was evaluated by quantitative real-time PCR (qPCR) [32].

The villous explants were cultured in 96-well plates overnight in 10% FBS medium at 37 °C with 5% CO_2_. Next, the villi were infected with TCTY (1 × 10^5^/villi) for 24 h. Then, the villi were treated for 72 h with OR and LHE (64 and 128 µg/mL), BZN (50 µM), and 5% FBS medium (control). After treatment, the supernatants were collected for further cytokine measurements, while the villi were stored at −80 °C until quantification of parasite load by qPCR. It is important to highlight that parasite load in placental tissue was assessed after four days of infection, in contrast to the three-day timeline used for the cell model. This difference is because infection takes longer in placental tissue than in cells because the villi consist of multiple layers, including cytotrophoblasts, syncytiotrophoblasts, mesenchyme, and blood vessels. This placental barrier makes it harder and slower for the parasite to infect the placenta compared to the quicker process in the in vitro cell model.

The DNA from villi was extracted using a DNA purification kit (Promega, São Paulo, SP, Brazil) and proteinase K (20 mg/mL). DNA quantification was performed using a NanoDrop spectrophotometer (Thermo Fisher Scientific, Wilmington, DE, USA) by measuring absorbances at wavelengths of 260 and 280 nm. DNA purity was evaluated by the ratio of absorbance readings at 260/280 nm, with values between 1.8 and 2.0 considered acceptable. For the qPCR reaction, the amount of genetic material was normalized to 100 ng of tissue DNA per reaction, and the GoTaq^®^ Master Mix kit (Promega) was used according to the manufacturer’s instructions. The primers used for DNA amplification were as follows: forward (5′-CGCAAACAGATATTGACAGAG-3′) and reverse (5′-TGTTCACACACTGGACACCAA-3′) [39]. Quantification of each sample was based on a standard curve prepared from serial two-fold dilutions starting at 100 ng of DNA, comprising six points. The results are expressed as TCTY equivalent to 100 ng of DNA tissue. Two independent experiments, with eight replicates, were carried out.

### 2.13. Cytokine Measurements

Human cytokines IL-4, IL-6, IL-8, IL-10, MIF, and TNF-α were quantified in the supernatants of BeWo cells and villous explants under different experimental conditions using a double-antibody sandwich enzyme-linked immunosorbent assay (ELISA), following the manufacturers’ protocol (BD Biosciences, San Jose, CA, USA or R&D Systems, Minneapolis, MN, USA). Cytokine levels in BeWo cells supernatants were expressed in pg/mL, whereas the villi values were normalized by the ratio between the concentration of cytokines (pg/mL) and the weight of each villous (mg), resulting in pg/mL/mg. The detection limits of each cytokine were as follows: IL-4, IL-10, TNF-α (7.8 pg/mL), IL-6 (4.7 pg/mL), IL-8 (3.12 pg/mL), and MIF (31.2 pg/mL).

### 2.14. Statistical Analysis

Statistical analysis was performed using GraphPad Prism Software version 8.0 (GraphPad Software Inc., San Diego, CA, USA, https://www.graphpad.com, Accessed on 15 June 2025). Data are presented as mean ± standard error of mean (SEM). Differences between groups were assessed by One-Way ANOVA test with Bonferroni’s multiple comparison post-test for the parametric data or Kruskal–Wallis’s test with Dunn’s multiple comparison post-test for the nonparametric data. A *p*-value of  <0.05 was considered statistically significant.

## 3. Results

### 3.1. Oleoresin and Leaf Hydroalcoholic Extract from C. multijuga Are Toxic to BeWo Cells and T. cruzi Trypomastigotes Only at Highest Concentrations

An MTT assay was performed to evaluate the effect of oleoresin and the leaf hydroalcoholic extract from *C. multijuga* on both BeWo cells and *T. cruzi* trypomastigote viability.

**Figure 1:**
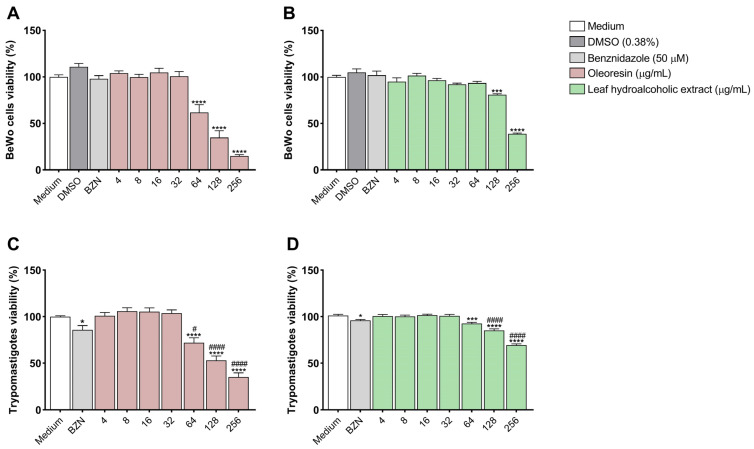
BeWo cell and trypomastigote viability treated with OR or LHE from *C. multijuga*. BeWo cells and *T. cruzi* trypomastigotes were treated with OR (**A**,**C**) or LHE (**B**,**D**) for 72 h and submitted to the MTT assay. Data is presented as percentage (%) of cell/trypomastigote viability compared to untreated cells/trypomastigotes (medium, 100% viability). Differences between groups were analyzed by One-Way ANOVA test with Bonferroni’s multiple comparisons post-test. Significant differences in relation to cells/trypomastigotes treated with medium (*p) or treated with benznidazole (^#^*p*). Three independent experiments, with eight replicates, were performed.

In BeWo cells, we observed that treatment with OR was cytotoxic at concentrations of 64, 128, and 256 µg/mL when compared to cells treated with medium (****p < 0.0001) (Figure 1A) with CC50 = 91.73 µg/mL, while LHE was cytotoxic only at the concentrations of 128 (***p = 0.0002) and 256 µg/mL (****p < 0.0001) compared to cells treated with medium (Figure 1B), presenting CC50 = 221.93 µg/mL.

In trypomastigotes, we observed that treatment with BZN (*p = 0.0179), OR at concentrations of 64, 128, and 256 µg/mL (****p < 0.0001) (Figure 1C), as well as LHE at 64 (***p = 0.0009) and 128 and 256 µg/mL (****p < 0.0001), were also cytotoxic compared to trypomastigotes treated with medium (Figure 1D). Interestingly, both OR and LHE showed higher cytotoxicity in trypomastigotes compared to BZN (^#^*p* < 0.05). OR showed CC50 = 150.3 µg/mL, while LHE had a CC50 > 256 µg/mL.

Based on these results, we chose two non-cytotoxic concentrations of each compound for BeWo cells to perform the next experiments. For oleoresin, 16 and 32 µg/mL were chosen, while 32 and 64 µg/mL were chosen for the leaf hydroalcoholic extract.

### 3.2. Oleoresin and Leaf Hydroalcoholic Extract from C. multijuga Decrease T. cruzi Intracellular Proliferation in BeWo Cells

To evaluate the impact of oleoresin and leaf hydroalcoholic extract on *T. cruzi* proliferation, we counted the number of infected cells and intracellular amastigotes.

**Figure 2:**
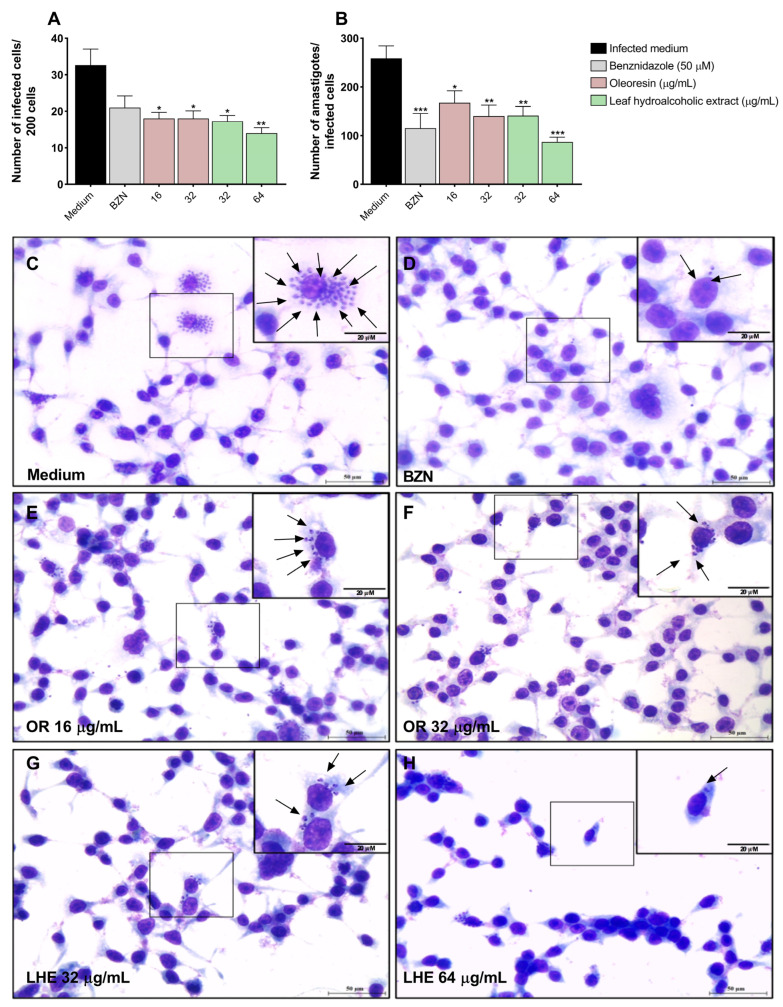
*T. cruzi* intracellular proliferation in BeWo cells treated with OR and LHE from *C. multijuga*. BeWo cells were infected with *T. cruzi* for 3 h and subsequently treated for 72 h with OR (16 and 32 µg/mL), LHE (32 and 64 µg/mL), BZN (50 µM), or FBS 5% medium. The number of infected cells (**A**) was analyzed under a light microscope to determine the infection index (the number of infected cells per 200 randomly observed cells) and the intracellular proliferation of *T. cruzi* (**B**) (the number of total amastigotes observed in the infected cells). Differences between groups were analyzed by the Kruskal–Wallis test with Dunn’s multiple comparisons post-test (**A**) or the One-Way ANOVA test with Bonferroni’s multiple comparisons post-test (**B**). Significant differences in relation to infected and untreated cells (medium) (*p). Three independent experiments with four replicates were performed. Representative images of BeWo cells infected with *T. cruzi* and untreated (**C**), treated with BZN (**D**), treated with OR 16 μg/mL (**E**) and 32 μg/mL (**F**), or LHE 32 μg/mL (**G**) and 64 μg/mL (**H**). Arrows indicate *T. cruzi* amastigotes in the cytoplasm of BeWo cells. Scale bar: 50 μm.

We observed that treatment with OR at concentrations of 16 (*p = 0.0262) and 32 μg/mL (*p = 0.0473), as well as LHE at concentrations of 32 (*p = 0.0343) and 64 μg/mL (**p = 0.0031) decreased the number of *T. cruzi*-infected cells compared to infected and untreated cells (infected medium) (Figure 2A). In addition, we observed that treatment with OR at concentrations of 16 (*p = 0.0185) and 32 μg/mL (**p = 0.0036), as well as LHE at concentrations of 32 (**p = 0.0038) and 64 μg/mL (***p = 0.0003), in addition to BZN (***p = 0.0008), decreased the number of amastigotes in infected cells compared to infected and untreated cells (infected medium) (Figure 2B).

Representative photomicrographs of BeWo cells (Figure 2C–H) are shown, where it is possible to observe several amastigotes (arrows) in the cytoplasm of infected and untreated BeWo cells (C) compared to cells infected and treated with BZN (D), OR 16 (E) and 32 μg/mL (F), and LHE 32 (G) and 64 μg/mL (H).

### 3.3. Oleoresin and Leaf Hydroalcoholic Extract from C. multijuga Decrease the Release of Trypomastigotes into the Supernatant of BeWo Cells

Since OR and LHE were affecting parasite proliferation, we also evaluated the effect of both compounds on trypomastigote release in the supernatant at different time points.

**Figure 3:**
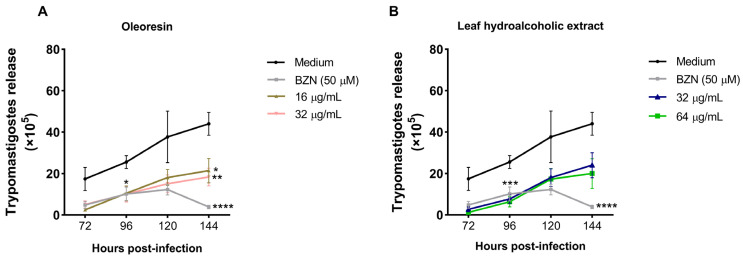
*T. cruzi* trypomastigote release in the supernatant of BeWo cells treated with (**A**) OR and (**B**) LHE from *C. multijuga*. The supernatant of BeWo cells infected and treated with OR (16 and 32 µg/mL), LHE (32 and 64 µg/mL), BZN (50 µM), or FBS 5% medium was collected after 72 h of treatment, as well as 96, 120, and 144 h after infection, and the trypomastigotes were counted in the Neubauer chamber. Data is presented as the number of trypomastigotes in the supernatant ×10^5^/mL. Differences between groups were analyzed by the Two-Way ANOVA test with Bonferroni’s multiple comparisons post-test. Significant differences in relation to infected and untreated cells (medium) (*p). Three independent experiments, with four replicates, were performed.

Regarding OR, we observed that both concentrations and BZN were able to decrease the release of trypomastigotes at all times compared to infected and untreated cells (medium), being statistically different at 96 (BZN: *p = 0.0139; 16 μg/mL: *p = 0.0166; 32 μg/mL: *p = 0.0157) and 144 h (BZN: ***p = 0.0004; 16 μg/mL: *p = 0.0388; 32 μg/mL: **p = 0.0072) (Figure 3A).

Concerning LHE, we observed that both concentrations also decreased the release of trypomastigotes at all times compared to infected and untreated cells (medium), being statistically different at 96 h (32 μg/mL: **p = 0.0036; 64 μg/mL: ***p = 0.0002), while BZN decreased at 96 (***p = 0.0005) and 144 h (***p = 0.0003) (Figure 3B).

### 3.4. Pretreatment of T. cruzi Trypomastigotes with Oleoresin and Leaf Hydroalcoholic Extract Compromises Its Ability to Invade and Proliferate in BeWo Cells

To evaluate whether pretreatment with both compounds had a direct effect on *T. cruzi* trypomastigotes, we performed an invasion and proliferation assay.

**Figure 4:**
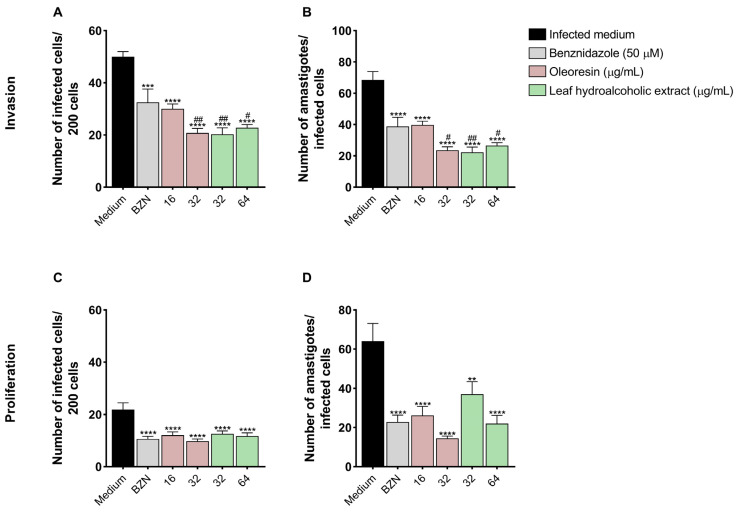
*T. cruzi* invasion and proliferation after pretreatment with OR and LHE. BeWo cells were infected with *T. cruzi* previously pretreated for 1 h with OR (16 and 32 µg/mL), LHE (32 and 64 µg/mL), BZN (50 µM), or FBS 5% medium, and then, the invasion (**A**,**B**) and proliferation (**C**,**D**) were analyzed. The percentage of invasion/proliferation of the parasite was analyzed under a light microscope to determine the infection index (the number of infected cells per 200 randomly observed cells) (**A**,**C**) and the invasion/proliferation of *T. cruzi* (the number of total amastigotes observed in the infected cells) (**B**,**D**). Differences between groups were analyzed by the One-Way ANOVA test with Bonferroni’s multiple comparisons post-test. Significant differences in relation to cells infected with untreated parasites (medium) (*p) or cells infected with BZN-treated parasites (^#^*p*). Two independent experiments, with four replicates, were performed.

In the invasion assay, we observed that pretreatment of the parasites with both concentrations of OR (****p < 0.0001) and LHE (****p < 0.0001), as well as BZN (***p = 0.0002), decreased the number of cells infected by *T. cruzi* compared to untreated parasites (medium), and both OR 32 μg/mL (^##^*p* < 0.0068) and LHE (32 μg/mL: ^##^*p* = 0.0002; 64 μg/mL: ^#^*p* < 0.0001) reduced more compared to BZN (Figure 4A). Also, both concentrations of OR and LHE, in addition to BZN, decreased the number of amastigotes compared to cells infected with untreated parasites (medium) (****p < 0.0001) and OR 32 μg/mL (^#^*p* < 0.0119) and LHE (32 μg/mL: ^##^*p* = 0.0073; 64 μg/mL: ^#^*p* < 0.0375) (Figure 4B).

Regarding the proliferation assay, we observed that pretreatment of parasites with both concentrations of OR (16 μg/mL: ***p = 0.0002; 32 μg/mL: ****p < 0.0001), LHE (****p < 0.0001), and BZN (****p < 0.0001) decreased the number of cells infected by *T. cruzi* compared to untreated parasites (medium) (Figure 4C). Furthermore, both concentrations of OR and LHE, as well as BZN, also decreased the number of amastigotes compared to cells infected with untreated parasites (medium) (****p < 0.0001) (Figure 4D).

### 3.5. Oleoresin and Leaf Hydroalcoholic Extract from C. multijuga Affect Both Trypomastigote and Amastigote Morphology

Since the invasion and proliferation of the parasite were being decreased by both compounds, we analyzed whether OR and LHE were affecting the trypomastigote and amastigote forms’ morphology by scanning electron microscopy (SEM) and transmission electron microscopy (TEM), respectively.

**Figure 5:**
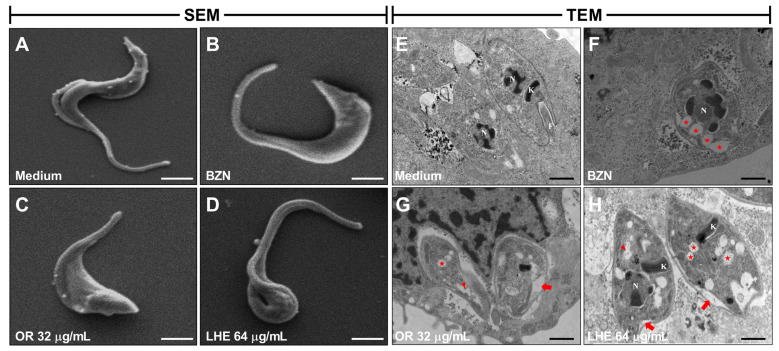
Effect of OR and LHE on *T. cruzi* trypomastigotes and amastigotes. *T. cruzi* trypomastigotes were pretreated for 1 h with OR (32 µg/mL), LHE (64 µg/mL), BZN (50 µM), or FBS 5% medium and then processed for scanning electron microscopy (**A**–**D**). Representative images of trypomastigotes treated with medium (**A**) with normal morphology, while BZN (**B**), OR (**C**), and LHE (**D**) displayed some abnormalities, such as swelling of the parasite’s body, rounding of the cell surface, shortening of the flagellum, and torsion. Scale bars: 2 µm. Transmission electron microscopy images (**E**–**H**) of *T. cruzi* amastigotes after 72 h of treatment with medium (**E**), BZN 50 µM (**F**), OR 32 µg/mL (**G**), and LHE 64 µg/mL (**H**). Thick arrow: membrane swelling; star: vacuolization; arrowhead: cytoplasm disorganization; N: nuclei; K: kinetoplast; F: flagellum. Scale bar: 0.5 µm.

Regarding the trypomastigote forms (Figure 5A–D), untreated parasites (A) displayed normal morphology, while BZN (B), OR (C), and LHE (D) treatment induced morphological alterations, such as swelling of the parasite body, rounding of the cell surface, shortening of the flagellum, and cellular torsion.

As for the amastigote forms (Figure 5E–H), untreated *T. cruzi* amastigotes (E) exhibited typical morphology, with an intact plasma membrane, flagellum (f), nucleus (n), and kinetoplast (k). In contrast, treatment with BZN (F) caused cytoplasmic vacuolization (indicated by a star), while both OR (G) and LHE (H) induced ultrastructural changes, such as membrane swelling (thick arrow), cytoplasmic disorganization (arrowhead), and additional vacuolization (star).

### 3.6. Oleoresin and Leaf Hydroalcoholic Extract from C. multijuga Modulate Reactive Oxygen Species in BeWo Cells

After observing that both compounds reduced *T. cruzi* proliferation and affected parasite morphology, we tested whether both OR and LHE modulate ROS production in infected and uninfected BeWo cells.

**Figure 6:**
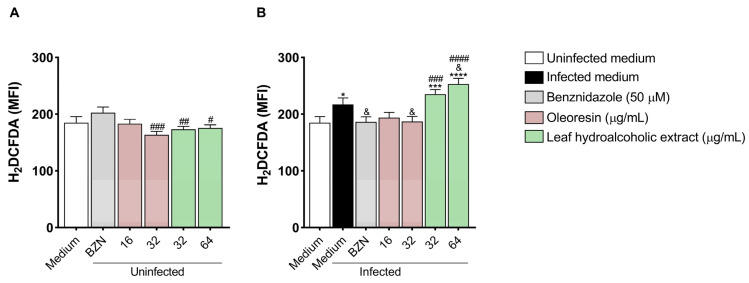
ROS production in BeWo cells. BeWo cells were infected or not infected with *T. cruzi* for 3 h and then treated or not treated for 72 h with OR (16 and 32 µg/mL), LHE (32 and 64 µg/mL), BZN (50 µM), or FBS 5% medium. The cells were incubated with a probe 2′,7′-dichlorodihydrofluorescein diacetate (H2DCF-DA), and ROS production was measured by fluorescence in a plate reader and expressed as median fluorescence intensity (MFI). Differences between groups were analyzed by One-Way ANOVA with Bonferroni’s multiple comparisons post-test. Significant differences in relation to uninfected and untreated cells (uninfected medium) (*p), infected and untreated cells (^&^*p*), and cells infected or uninfected and treated with BZN (^#^*p*) (**A**,**B**). Three independent experiments, with eight replicates, were performed.

In uninfected cells, we observed that the concentration of 32 μg/mL of OR (^###^*p* = 0.0004) and both concentrations of LHE (32 μg/mL: ^##^*p* = 0.0078; 64 μg/mL: ^#^*p* = 0.0133) induced lower ROS production compared to cells treated with BZN (Figure 6A).

In the presence of infection, BeWo cells infected and untreated (infected medium, *p = 0.0232) or infected and treated with both concentrations of LHE (32 μg/mL: ***p = 0.0006; 64 μg/mL: ****p < 0.0001) increased ROS production compared to uninfected and untreated cells (uninfected medium) (Figure 6B). In addition, cells infected and treated with BZN (^&^*p* = 0.0258) and OR (32 μg/mL) (^&^*p* = 0.0276) decreased ROS production compared to cells infected and untreated (infected medium), whereas LHE (64 μg/mL) increased (^&^*p* = 0.0125) (Figure 6B). Furthermore, both concentrations of LHE (32 μg/mL: ^###^*p* = 0.0006; 64 μg/mL: ^####^*p* < 0.0001) had a higher ROS production compared to cells infected and treated with BZN (Figure 6B).

### 3.7. Oleoresin and Leaf Hydroalcoholic Extract from C. multijuga Increase IL-4, IL-8, IL-10, and TNF-α Production in BeWo Cells

We also tested whether OR and LHE would interfere with the host immune response through cytokine production.

**Figure 7:**
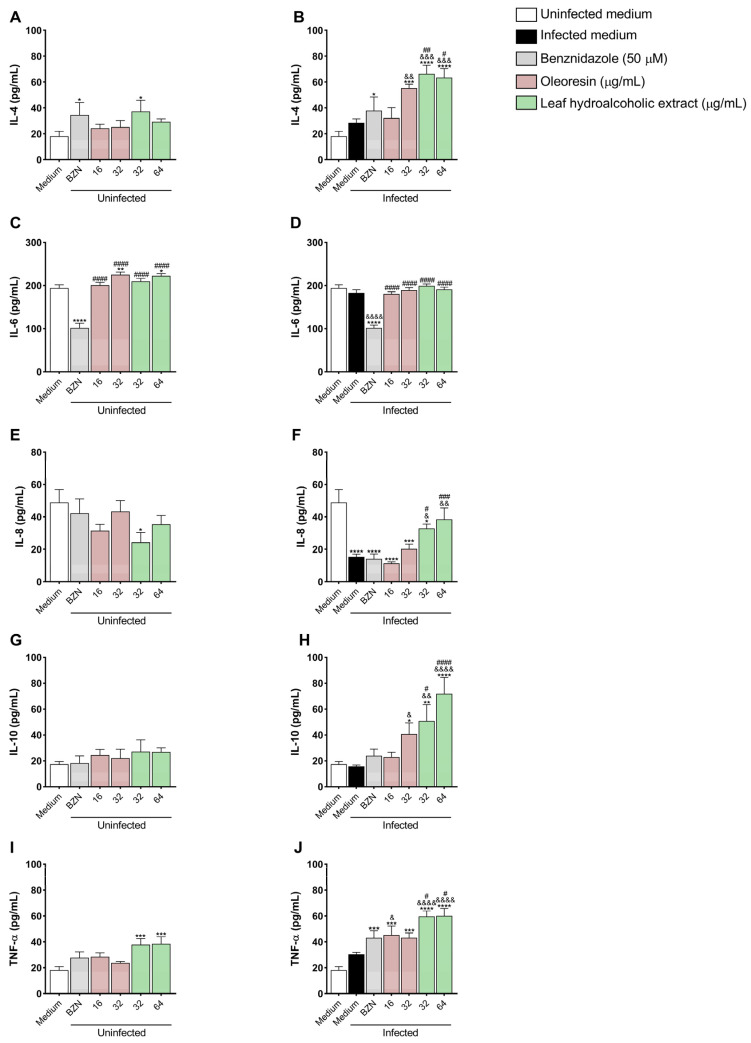
Cytokine production in BeWo cells. BeWo cells were infected or not infected with *T. cruzi* for 3 h and then treated or not treated for 72 h with OR (16 and 32 µg/mL), LHE (32 and 64 µg/mL), BZN (50 µM), or FBS 5% medium. The supernatant was submitted to an ELISA assay for the measurement of IL-4 (**A**,**B**), IL-6 (**C**,**D**), IL-8 (**E**,**F**), IL-10 (**G**,**H**), and TNF-α (**I**,**J**). Data are presented in pg/mL according to the standard curve for each cytokine. Differences between groups were analyzed by One-Way ANOVA with Bonferroni’s multiple comparisons. Significant differences in relation to uninfected and untreated cells (uninfected medium) (*p), infected and untreated cells (^&^*p*), and cells infected or not infected and treated with BZN (^#^*p*). Three independent experiments, with three replicates, were performed.

IL-4: Uninfected cells treated with BZN (*p = 0.0432) and LHE at 32 μg/mL (*p = 0.0133) (Figure 7A), as well as infected cells treated with BZN (*p = 0.0448), OR at 32 μg/mL (***p = 0.0001), and both concentrations of LHE (****p < 0.0001) (Figure 7B) showed increased IL-4 production compared to uninfected untreated cells (uninfected medium). Infected cells treated with OR at 32 μg/mL (^&&^*p* = 0.0077), and both concentrations of LHE (32 μg/mL: ^&&&^*p* = 0.0003; 64 μg/mL: ^&&&^*p* = 0.0007) exhibited increased IL-4 levels compared to infected untreated cells (infected medium) (Figure 7B). In addition, both LHE concentrations (32 μg/mL: ^##^*p* = 0.0051; 64 μg/mL: ^#^*p* = 0.0109) induced more IL-4 production compared to infected cells treated with BZN (Figure 7B).

IL-6: Uninfected cells treated with BZN (****p < 0.0001) showed decreased IL-6 levels, while OR at 32 μg/mL (**p = 0.0059) and LHE at 64 μg/mL (*p = 0.0109) showed increased IL-6 production compared to uninfected untreated cells (uninfected medium) (Figure 7C). In addition, both OR and LHE (^####^*p* < 0.0001) induced more IL-6 compared to those treated with BZN (Figure 7C). Infected cells treated with BZN showed decreased IL-6 production compared to uninfected untreated cells (uninfected medium) (****p < 0.0001) and infected untreated cells (infected medium) (^&&&&^*p* < 0.0001). In addition, infected cells treated either with OR or LHE induced more IL-6 (^####^*p* < 0.0001) compared to those infected cells treated with BZN (Figure 7D).

IL-8: In the absence of the parasite, only 32 μg/mL of LHE (*p = 0.0165) showed decreased IL-8 production compared to uninfected untreated cells (uninfected medium) (Figure 7E). Also, infected untreated cells (****p < 0.0001), or treated with BZN (****p < 0.0001), OR (16 μg/mL: ****p < 0.0001; 32 μg/mL: ***p = 0.0001) or LHE at 32 μg/mL (*p = 0.0389) exhibited decreased IL-8 levels compared to uninfected untreated cells (uninfected medium) (Figure 7F). In addition, both concentrations of LHE induced more IL-8 compared to only infected cells (32 μg/mL: ^&^*p* = 0.0263; 64 μg/mL: ^&&^*p* = 0.0011), as well as those infected and treated with BZN (32 μg/mL: ^#^*p* = 0.0148; 64 μg/mL: ^###^*p* = 0.0004) (Figure 7F).

IL-10: Infected cells treated with OR at 32 μg/mL (*p = 0.0165) and both concentrations of LHE (32 μg/mL: **p = 0.003; 64 μg/mL: ****p < 0.0001) induced more IL-10 compared to uninfected untreated cells (uninfected medium), as well as infected untreated cells (infected medium) (OR 32 μg/mL: ^&^*p* = 0.0107; LHE 32 μg/mL: ^&&^*p* = 0.0026; LHE 64 μg/mL: ^&&&&^*p* < 0.0001) (Figure 7H). In addition, LHE induced more IL-10 compared to those infected cells treated with BZN (32 μg/mL: ^#^*p* = 0.0150; 64 μg/mL: ^####^*p* < 0.0004) (Figure 7H).

TNF-α: Uninfected cells treated with LHE (32 μg/mL: ***p = 0.001; 64 μg/mL: ***p = 0.0007) showed increased TNF-α levels compared to uninfected untreated cells (uninfected medium) (Figure 7I). In addition, infected cells treated with BZN (***p = 0.003), OR (16 μg/mL: ***p = 0.0002; 32 μg/mL: ****p = 0.0004), or LHE (****p < 0.0001) induced more TNF-α compared to uninfected untreated cells (uninfected medium) (Figure 7J). Also, treatment with OR at 16 μg/mL (^&^*p* = 0.0323) and LHE (^&&&&^*p* < 0.0001) exhibited increased TNF-α production compared to infected untreated cells (infected medium), and LHE induced more TNF-α compared to those infected cells treated with BZN (32 μg/mL: ^#^*p* = 0.0112; 64 μg/mL: ^#^*p* = 0.0121) (Figure 7J).

MIF: Levels were below the detection threshold in all experimental conditions.

### 3.8. Oleoresin and Leaf Hydroalcoholic Extract from C. multijuga Reduce T. cruzi Infection in Human Placenta Explants

Following the assessment in the in vitro cell model, we evaluated whether OR and LHE could reduce *T. cruzi* infection using human placenta explants.

**Figure 8:**
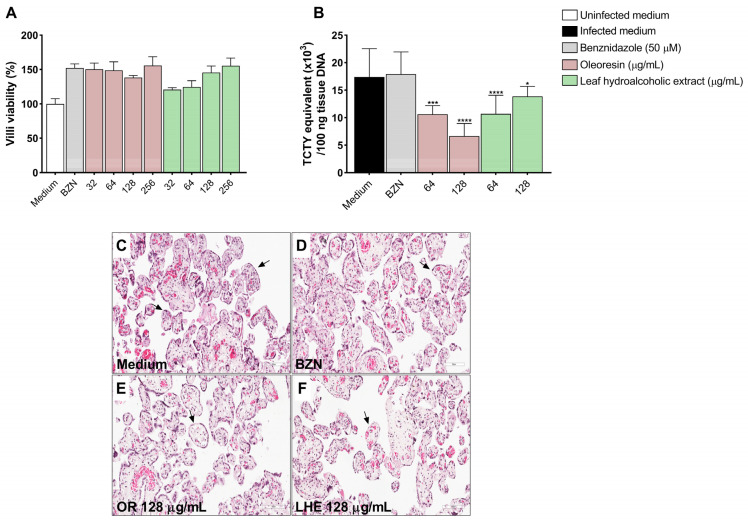
*T. cruzi* DNA content in human placenta explants. Human placental explants were treated for 72 h with OR, LHE, BZN (50 µM), or FBS 5% medium and submitted to an MTT assay (**A**). Data are presented as the percentage (%) of villi viability in relation to untreated villi (untreated medium). Two independent experiments, with eight replicates, were performed. In addition, the villi were infected with *T. cruzi* and treated for 72 h with OR (64 and 128 µg/mL), LHE (64 and 128 µg/mL), BZN (50 µM), and FBS 5% medium and then processed to PCR for quantification of *T. cruzi* DNA content (**B**). Data are presented as TCTY equivalent per 100 ng of DNA tissue. Representative images are shown of untreated villi (**C**), treated with BZN (50 µM) (**D**), OR (128 µg/mL) (**E**), and LHE (128 µg/mL) (**F**). Histological sections are stained by Harris hematoxylin and eosin, where it is possible to see no damage in the syncytiotrophoblast layer (arrow). Scale bar: 100 µm. Differences between groups were analyzed by One-Way ANOVA with Bonferroni’s multiple comparisons. Significant differences in relation to villi infected and untreated (medium) (*p). Two independent experiments, with eight replicates, were performed.

As measured by viability assays, no cytotoxic effects were observed at any concentrations of OR and LHE on villous explants (Figure 8A), and histological evaluation showed no evidence of tissue damage (Figure 8C–F). Treatment with OR (64 μg/mL: ***p = 0.0001; 128 μg/mL: ****p < 0.0001) and LHE (64 μg/mL: ****p < 0.0001; 128 μg/mL: *p = 0.0244) significantly reduced parasite DNA content compared to infected and untreated explants (Figure 8B).

### 3.9. Oleoresin Increases IL-6 and Decreases TNF-α Production in Human Placenta Explants

Cytokine production was evaluated in villous explants following infection and/or treatment with OR and LHE.

**Figure 9:**
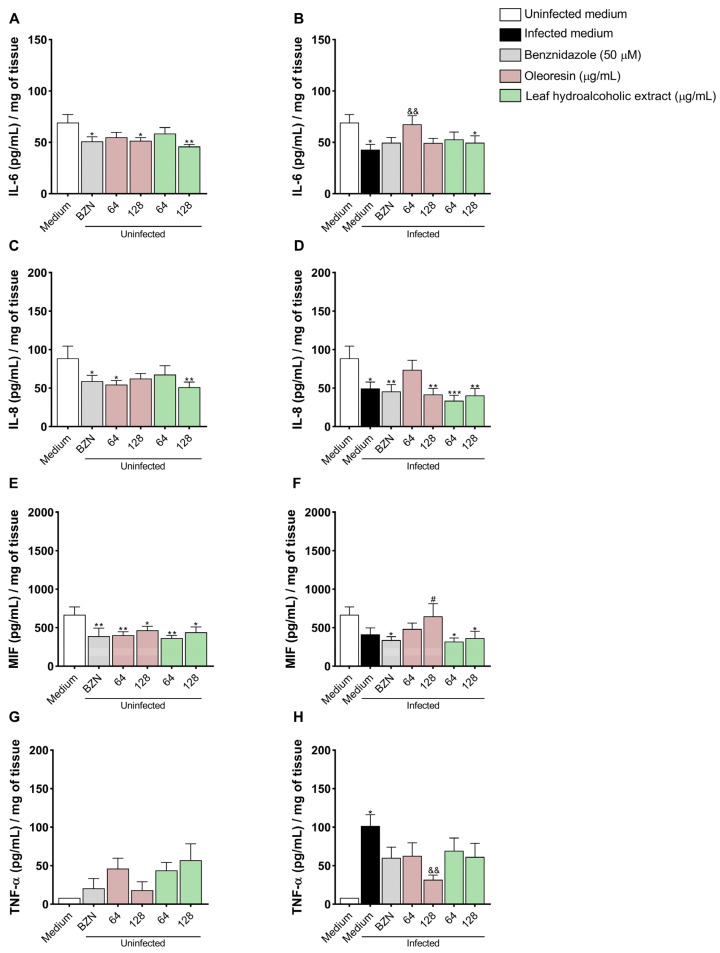
Cytokine production in human placenta explants. BeWo cells were infected or not infected with *T. cruzi* for 3 h and then treated or not treated for 72 h with OR (64 and 128 µg/mL), LHE (64 and 128 µg/mL), BZN (50 µM), or FBS 5% medium. The supernatant was submitted to an ELISA assay for the measurement of IL-6 (**A**,**B**), IL-8 (**C**,**D**), MIF (**E**,**F**), and TNF-α (**G**,**H**). Data are presented in pg/mL/mg of tissue. Differences between groups were analyzed by One-Way ANOVA with Bonferroni’s multiple comparisons post-test (**A**–**F**,**H**) or the Kruskal–Wallis test with Dunn’s multiple comparisons post-test (**G**). Significant differences in relation to uninfected and untreated cells (uninfected medium) (*p), infected and untreated cells (^&^*p*), and cells infected or not infected and treated with BZN (^#^*p*). Two independent experiments, with eight replicates, were performed.

IL-6: Uninfected villi treated with BZN (*p = 0.0188), OR at 128 μg/mL (*p = 0.0146), and LHE at 128 μg/mL (**p = 0.0026) exhibited significantly reduced IL-6 production compared to untreated and uninfected villi (uninfected medium) (Figure 9A). Additionally, infected and untreated villi (*p = 0.0108) and those treated with LHE at 128 μg/mL (*p = 0.0481) showed decreased IL-6 production compared to untreated uninfected villi (uninfected medium), whereas infected villi treated with OR at 64 µg/mL (^&&^*p* = 0.0083) exhibited increased IL-6 levels compared to infected untreated villi (infected medium) (Figure 9B).

IL-8: Uninfected villi treated with BZN (*p = 0.0435), OR at 64 μg/mL (*p = 0.0177), and LHE at 128 μg/mL (**p = 0.0097) showed reduced IL-8 production compared to untreated uninfected villi (uninfected medium) (Figure 9C). Similarly, infected and untreated villi (*p = 0.0109), or those treated with BZN (**p = 0.0065), OR at 128 μg/mL (**p = 0.0025), and LHE (64 μg/mL: ***p = 0.0003; 128 μg/mL: **p = 0.0013) also exhibited reduced IL-8 production compared to untreated uninfected villi (uninfected medium) (Figure 9D).

MIF: Uninfected villi treated with BZN (**p = 0.0089), OR (64 μg/mL: **p = 0.0092; 128 μg/mL: *p = 0.0424), and LHE (64 μg/mL: **p = 0.0032; 128 μg/mL: *p = 0.0273) exhibited decreased MIF levels compared to untreated uninfected villi (uninfected medium) (Figure 9E). Infected villi treated with BZN (*p = 0.03) and LHE (64 μg/mL: *p = 0.0159; 128 μg/mL: *p = 0.0349) also showed reduced MIF levels compared to untreated uninfected villi. However, infected villi treated with LHE at 128 μg/mL produced more MIF than those with BZN (^#^*p* = 0.0317) (Figure 9F).

TNF-α: Only infected untreated villi (infected medium, *p = 0.0384) showed increased TNF-α production compared to untreated uninfected villi. In contrast, infected villi treated with OR at 128 μg/mL (^&&^*p* = 0.0036) exhibited decreased TNF-α levels compared to infected untreated villi (Figure 9H).

IL-4 and IL-10: Levels were below the detection threshold in all experimental conditions.

## 4. Discussion

The limitation of benznidazole and nifurtimox for the treatment of congenital Chagas disease has arisen since they can cause side effects for the pregnant woman, with possible teratogenic effects, as well as the appearance of resistant parasites [40,41], making the search for novel drugs that are effective and safe an urgent need and of great importance. Plant-based compound studies have drawn attention as promising sources for novel drug discovery. Therefore, this study investigated the effects of oleoresin and leaf hydroalcoholic extract from *C. multijuga* on *T. cruzi* infection at the human maternal–fetal interface.

To be considered a potential new therapeutic agent against infectious diseases, it must not only control the infection but also exhibit low toxicity for the host. To address these challenges, we first evaluated the impact of OR and LHE on BeWo cell viability. Our results show that BeWo cells treated with OR and LHE exhibited reduced cell viability only at the highest concentrations. In agreement with our results, [22] sought to evaluate the genotoxicity of oleoresin from *C. multijuga* in Swiss mouse hepatocytes, and it was seen that it only caused some damage at the highest concentration. In a recent study from our group, both OR and LHE of *C. multijuga* were also toxic to BeWo and HTR-8/SVneo cells only at higher concentrations after 24 h of treatment [26], while in our current study, we analyzed after 72 h.

Furthermore, we evaluated the effect of OR and LHE on *T. cruzi* trypomastigote viability. Like the results observed in cells, both compounds exhibited cytotoxicity only at the highest concentrations (64, 128, and 256 µg/mL). Notably, the reduction in trypomastigote viability caused by OR and LHE was greater than that achieved with benznidazole. Although conventional treatment is more effective during the acute phase of the disease (around 60%), when trypomastigotes circulate in the bloodstream, studies have shown that the parasite has developed resistance mechanisms to BZN [42,43,44]. This underscores the need for new therapeutic strategies that are effective, especially in the acute phase of infection.

Our data shows that both compounds were able to decrease *T. cruzi* intracellular proliferation in BeWo cells. Corroborating our findings, Izumi and colleagues [45] demonstrated that terpenes found in oleoresins of *Copaifera* spp. promoted changes in oxidative metabolism, induced autophagy, and decreased the proliferation of the replicative forms of *T. cruzi*, especially amastigotes. In another study, Izumi and colleagues [31] evaluated the effect of oleoresin from eight species of *Copaifera*, including *C. multijuga*, and observed strong inhibitory effects on all life stages of the parasite. Additionally, oleoresin and kaurenoic acid from *C. martii* were shown to significantly reduce infection rates and the proliferation of *T. cruzi* amastigotes in murine peritoneal macrophages and HeLa cells [38]. Recently, our group reported that ORs from five different species of *Copaifera* controlled *T. cruzi* invasion, replication, and release in BeWo cells [32], further highlighting the trypanocidal potential of these compounds.

Once inside the host cell, *T. cruzi* amastigotes replicate in the cytoplasm and then differentiate back into trypomastigotes, which are released into the extracellular space to infect new cells and spread through the bloodstream. Since treatment with OR and LHE decreased the number of amastigotes in BeWo cells, we investigated whether this would also affect the differentiation and subsequent release of trypomastigotes. Although no statistical differences were observed at 72 and 120 h, our data showed a trend toward reduced trypomastigote release into the supernatant, suggesting a direct action of these compounds on the parasite that may compromise its ability to differentiate, lyse the host, infect new cells, and spread throughout the organism.

To test this hypothesis, we performed invasion and proliferation assays after pre-treating trypomastigotes for 1 h. Our data shows that pretreated parasites exhibited a reduced ability to invade and proliferate within BeWo cells. A recent study suggested that natural compounds may target different structures in *T. cruzi*, and it is hypothesized that mitochondria are strategic sites to induce parasite death [46]. To evaluate whether OR and LHE from *C. multijuga* affect parasite morphology, we analyzed trypomastigote forms pretreated for 1 h using a scanning electron microscope and observed that both compounds induced morphological alterations in the parasite body, such as shortening of the body and flagellum and torsion of the parasite, which may explain its difficulty in invading and proliferating inside the cell. Additionally, transmission electron microscopy of amastigotes treated for 72 h revealed further ultrastructural damage, such as cytoplasmic disorganization, membrane swelling, and vacuolization, supporting the observed reduction in parasite proliferation. Previous studies have reported similar effects of *Copaifera* spp. on other protozoa, such as membrane fusion in *T. gondii*, compromising its replicative capacity [25], rupture of the plasma membrane, retraction of the flagellum, and rounding of *Leishmania* spp. [29,47], as well as rupture of the plasma membrane and intracellular vacuole-like structures in *T. cruzi* [31,45]. Also, [32] showed that both *T. cruzi* amastigotes and trypomastigotes treated with *Copaifera* spp. undergo morphological modifications, such as disruption of the amastigote’s plasma membrane, kinetoplast swelling, and membrane shedding. These findings are important for the discovery of organelles and target molecules of the parasite to evaluate the possible therapeutic use of these compounds, and further studies are needed to discover which molecules present in these compounds have antiparasitic properties.

After demonstrating the direct effect of *C. multijuga* on *T. cruzi* trypomastigotes and amastigotes, we next evaluated whether OR and LHE could modulate the host immune response. We observed that *T. cruzi* infection increased ROS production compared to uninfected cells. Although the mechanisms are not fully understood, it is known that elevated ROS can promote *T. cruzi* infection and contribute to Chagas disease pathology [48,49]. Interestingly, treatment with BZN and the highest concentration of OR decreased ROS production compared to infected cells, while the highest concentration of LHE (64 μg/mL) increased it. Although both compounds are derived from the same plant species, these differences may be attributed to their distinct chemical composition, since ORs have more diterpenes and sesquiterpenes, while leaf extracts have more flavonoids and galloylquinic acids [50,51,52], so it is plausible that they can trigger different intracellular mechanisms.

Oleoresins from *Copaifera* spp. are known for their anti-inflammatory properties [53], which may help reduce ROS production. Supporting this, Teixeira and colleagues [25] observed that four *Copaifera* species reduced ROS levels in *T. gondii*-infected BeWo cells while also limiting parasite proliferation. Similarly, Kian and colleagues [38] demonstrated that *C. martii* oleoresin decreased ROS production in *T. cruzi*-infected macrophages and helped control parasite growth, highlighting that one of the antiparasitic mechanisms promoted by *Copaifera* spp. is through the induction of an anti-inflammatory response, which is important for a successful pregnancy, since an exacerbated inflammatory response may lead to a gestational imbalance, leading to miscarriage. In addition, in the context of *T. cruzi* infection, this decrease in ROS can contribute to controlling parasite proliferation. Although ROS has an important role in controlling pathogen infection, the parasite has developed mechanisms to overcome this response and survive within cells, and this oxidative stress induces cellular damage that contributes to parasite replication during the acute infection phase [54]. On the other hand, LHE increased ROS production compared to cells that were only infected. It is known that ROS are produced mainly in mitochondria and, at basal levels, play essential roles in cellular physiology, but in high concentrations, these molecules can induce oxidative stress, causing the oxidation of macromolecules, mainly lipids, proteins, and DNA, which can trigger cell death [55]. In this sense, Lazarin-Bidóia and colleagues [56] demonstrated that eupomatenoid-5 (isolated from the leaves of *Piper regnellii* var. *pallescens*) induced an oxidative imbalance in the three evolutionary forms of *T. cruzi*, especially trypomastigotes, shown by a downregulation in the activity of trypanothione reductase and an upregulation of ROS, which are key events in the induction of parasite death by several pathways, including apoptosis, necrosis, and autophagy. Thus, it is possible to suggest that the increase in ROS production induced by LHE may partially contribute to the control of *T. cruzi* proliferation in BeWo cells, but the trypanocidal effect promoted by LHE is more related to a direct action of this compound on the parasite than to the positive modulation of ROS.

Regarding cytokine production, OR treatment increased levels of IL-4, IL-10, and TNF-α, while LHE increased levels of IL-4, IL-8, IL-10, and TNF-α in BeWo cells. Santiago and colleagues [57] showed that oleoresins from different *Copaifera* species, including *C. multijuga*, can increase the production of IL-10 and TNF-α in human monocytes. Additionally, Kian and colleagues [38] demonstrated that *C. martii* oleoresins and the isolated compound kaurenoic acid reduced *T. cruzi* amastigote replication in macrophages without triggering an exacerbated pro-inflammatory response. This was evidenced by the downregulation of ROS and upregulation of IL-10, which is consistent with our findings. Given that BeWo cells are not classical immune cells, cytokine results derived from this model should be interpreted within the context of trophoblast biology.

*T. cruzi* is known to induce TNF-α production in human placenta, which can disrupt the placental barrier and contribute to low birth weight [58]. However, a study showed that elevated TNF-α levels are associated with the replication stage of the parasite within the chorionic villus (72 h after infection), which would hinder its transformation into a trypomastigote and consequently limit infection of the fetus [59]. This increase in TNF-α production induced by LHE can also induce the epithelial turnover [13], an important mechanism to limit the parasite’s congenital transmission [60]. Despite the high production of the pro-inflammatory cytokines TNF-α and IL-8 induced by OR and LHE, there was a simultaneous increase in IL-4 and IL-10, both anti-inflammatory cytokines, which would counterbalance the immune response towards a more anti-inflammatory environment, protecting the placenta and the fetus from an exacerbated pro-inflammatory immune response and maintaining a tolerogenic environment during pregnancy, in addition to controlling the *T. cruzi* proliferation.

Subsequently, we evaluated whether OR and LHE could control the infection in an ex vivo model using third-trimester human placenta explants. We observed that both compounds were non-cytotoxic, maintained villous morphology, and effectively controlled *T. cruzi* infection. Previous studies have demonstrated the potential of different *Copaifera* spp. to control *T. cruzi* and *T. gondii* infection in human placentas [25,26,27,32]. Interestingly, treatment with BZN did not reduce *T. cruzi* proliferation in our model. In agreement with our findings, Rojo and colleagues [61] showed that placenta explants infected with *T. cruzi* (DM28c strain) and treated with BZN (2, 20, and 200 µM) did not impair parasite infection, although BZN did prevent parasite-induced tissue damage. Conversely, Lima Júnior and colleagues [32] demonstrated that the same concentration used in this study was able to reduce parasitism. Nevertheless, despite using the same strain, the authors evaluated it after 48 h of treatment, while ours was at 72 h, so it is possible that BZN can control the proliferation of *T. cruzi* in earlier stages of infection, while in later stages, the parasite recovers its proliferative capacity. However, further studies are needed to verify, in terms of concentration, cytotoxicity, and control of parasitism against different strains, the efficacy and reliability of BZN in the treatment of congenital Chagas disease.

Regarding cytokine production in placenta explants, similar to the observations in BeWo cells, the parasite decreased IL-6 and IL-8 production, along with a trend toward reduced MIF production while increasing TNF-α levels. Other studies showed that *T. cruzi* downmodulates IL-6 and upregulates TNF-α, which can eliminate the parasite but at the same time cause tissue damage in high concentrations [12,62]. However, treatment with OR reversed this phenomenon, with upregulation of IL-6, IL-8, and MIF and downregulation of TNF-α. These findings suggest that OR helps restore the balance of key cytokines important for a healthy pregnancy while also limiting the overproduction of TNF-α, which, in excess, can increase the risk of pregnancy complications [63]. A similar TNF-α reduction induced by *Copaifera* ORs has been observed in human placentas infected by *T. gondii* [25,27] and *T. cruzi* [32].

## 5. Conclusions

Considering our results and the limited alternative treatments for CCD, we conclude that oleoresin and leaf hydroalcoholic extract of *Copaifera multijuga* effectively controlled the invasion, proliferation, and dissemination of *T. cruzi* in BeWo cells and human placenta explants. This effect appears to be associated with both direct action on the parasite and the modulation of ROS, pro-inflammatory, and anti-inflammatory cytokines. These findings highlight the potential of these natural compounds as promising therapeutic candidates for the treatment of congenital Chagas disease. However, further studies are needed to evaluate which bioactive molecules present in these compounds have antiparasitic activity.

## Data Availability

The original contributions presented in this study are included in the article. Further inquiries can be directed to the corresponding author.
